# A de novo frameshift pathogenic variant in *TBR1* identified in autism without intellectual disability

**DOI:** 10.1186/s40246-020-00281-5

**Published:** 2020-09-18

**Authors:** Laurie-Anne Sapey-Triomphe, Julie Reversat, Gaëtan Lesca, Nicolas Chatron, Marina Bussa, Sylvie Mazoyer, Christina Schmitz, Sandrine Sonié, Patrick Edery

**Affiliations:** 1 Lyon Neuroscience Research Center, Brain Dynamics and Cognition team, INSERM UMRS 1028, CNRS UMR 5292, Université Claude Bernard Lyon 1, Université de Lyon, F-69000 Lyon, France; 2 Laboratory of Experimental Psychology, Department of Brain and Cognition, Leuven Brain Institute, KU Leuven, Leuven, Belgium; 3 Lyon Hospitals, Genetics Service and National Reference Centre for Developmental Anomalies, Lyon, France; 4 Lyon Neuroscience Research Center, Genetics of Neurodevelopment team, INSERM UMRS 1028, CNRS UMR 5292, Université Claude Bernard Lyon 1, Université de Lyon, F-69000 Lyon, France; 5 Centre de Ressource Autisme Rhône-Alpes, Centre Hospitalier Le Vinatier, Bron, France; 6 Hôpital Saint-Jean-de-Dieu, Lyon, France

## Abstract

**Background:**

In order to be able to provide accurate genetic counseling to patients with Autism Spectrum Disorder (ASD), it is crucial to identify correlations between heterogeneous phenotypes and genetic alterations. Among the hundreds of de novo pathogenic variants reported in ASD, single-nucleotide variations and small insertions/deletions were reported in *TBR1*. This gene encodes a transcription factor that plays a key role in brain development. Pathogenic variants in *TBR1* are often associated with severe forms of ASD, including intellectual disability and language impairment.

**Methods:**

Adults diagnosed with ASD but without intellectual disability (diagnosis of Asperger syndrome, according to the DSM-IV) took part in a genetic consultation encompassing metabolic assessments, a molecular karyotype and the screening of a panel of 268 genes involved in intellectual disability, ASD and epilepsy. In addition, the patient reported here went through a neuropsychological assessment, structural magnetic resonance imaging and magnetic resonance spectroscopy measurements.

**Results:**

Here, we report the case of a young adult male who presents with a typical form of ASD. Importantly, this patient presents with no intellectual disability or language impairment, despite a de novo heterozygous frameshift pathogenic variant in *TBR1*, leading to an early premature termination codon (c.26del, p.(Pro9Leufs*12)).

**Conclusion:**

Based on this case report, we discuss the role of TBR1 in general brain development, language development, intellectual disability and other symptoms of ASD. Providing a detailed clinical description of the individuals with such pathogenic variants should help to understand the genotype-phenotype relationships in ASD.

## Background

Autism Spectrum Disorder (ASD) is characterized by difficulties in social interactions and communication, together with restricted repertories of interests and behaviors and an atypical sensory sensitivity [1]. The prevalence of this neurodevelopmental disorder is around 1% [2]. The previous version of the Diagnostic and Statistical Manual of Mental Disorders (DSM-IV) [3] distinguished subtypes of ASD, whereas the current version (DSM-V) considers autism as a spectrum [1]. This notion of spectrum highlights the diversity of symptoms and severity in ASD (e.g., individuals with or without intellectual disability, verbal or non-verbal).

Even though major advances have been done, the genetic architecture of ASD is not fully characterized yet. ASD presents with genetic heterogeneity, and the causes lie either on genetic aberrations, i.e., large chromosomal rearrangements, copy number variants (CNVs) or single-nucleotide variants (SNVs), or on possible interactions between genetic, epigenetic and environmental factors [4]. Hundreds of gene variants with highly variable risk effects have been identified in ASD [5], including common variants identified recently through a very large genome-wide association study [6]. These variants include single-nucleotide variations (SNV), small insertions and deletions (indels), and CNV with loss/gain of thousands of nucleotides, which can either be inherited or de novo. Rare genetic variants would be causal in 10 to 30% of individuals with ASD [5], while the contribution of cumulated common inherited variants is estimated at around 50% [7–9]. The contribution of common variants would be more prominent in cases of “high-functioning” ASD, such as Asperger syndrome, while de novo variants are more frequently reported in ASD cases with intellectual disability [6]. An increased rate of de novo pathogenic variants is observed in ASD probands, as an average of around 74 de novo SNV and 13 de novo CNV are found in the genome of individuals with ASD [10]. Yet, each de novo pathogenic variants encountered in ASD account for less than 1% of the cases.

As recently highlighted [11], reports of ASD variants should include a comprehensive clinical description in order to contribute to a better understanding of the genotype-phenotype relationships in ASD. Especially, this correlation is missing in the less severe forms of ASD, such as cases of ASD without intellectual disability (e.g., Asperger syndrome in the DSM-IV [3]). Previous studies have evidenced genetic variations in individuals with Asperger syndrome in genes such as *OXTR* [12], *STX1A* [13], *ARNT2* [14], or *GABRB3* [15]. Providing genotype-phenotype characterizations in the forms of ASD without intellectual disability may shed light on the mechanisms underlying the symptoms of ASD (e.g., independently from genetic variations leading to intellectual disability).

Here, we investigated the genetics of ASD without intellectual disability. For this purpose, patients diagnosed with Asperger syndrome (DSM-IV [3]), who were part of a neuroimaging study, were given the possibility to have a genetic consultation. This consultation encompassed metabolic assessments, a molecular karyotype and the screening of a panel of 268 genes involved in intellectual disability, ASD, and epilepsy. Among the four adult participants with ASD who volunteered for the genetic consultation, one of them was identified with a de novo heterozygous truncating pathogenic variant in *TBR1.* The present article reports the case study of this patient.

## Methods

### Sample characteristics

The patient was a 28-year-old man with ASD, diagnosed before 2013 with Asperger syndrome according to the DSM-IV [3]. According to the Declaration of Helsinki, the patient provided written informed consent before the MRI acquisition, which obtained approval from the local ethics committee (South East IV Committee for the Protection of Persons), as well as written informed consents for genetic testing. The parents of the patient also provided written informed consents for genetic testing.

### Diagnostic tools

The patient underwent the Autism Diagnostic Observation Schedule (ADOS) [16] to measure the symptom severity and the WAIS-IV to measure his intellectual quotient [17]. His parents took the Autism Diagnostic Interview-Revised (ADI-R) [18] investigating the behavior of their son in the areas of social interactions, communication, and restricted and stereotyped behaviors during development, and the Vineland Adaptive Behavior Scale (Vineland-II) [19] assessing adaptive behaviors in the areas of communication, socialization and daily life.

### Self-administered questionnaires

The patient filled in two online questionnaires: the Autism-spectrum Quotient (AQ) quantifying autistic traits [﻿20﻿] (French version: [﻿21﻿]), and the Glasgow Sensory Questionnaire (GSQ) [﻿22﻿] (French version: [23]) assessing his profile of hyper and hyposensitivity.

### Neuroimaging assessment

The patient was involved in a functional MRI study, which included the acquisition of a high-resolution whole brain T1-weighted anatomical image on a 3-Tesla Magnetom Prisma magnetic resonance scanner, with a 64-channel head-neck coil (MPRAGE sequence, TR = 3500 ms, TE = 3.42 ms, slice number = 192, voxel size = 0.9 × 0.9 × 0.9 mm^3^). Single voxel magnetic resonance spectra were acquired in the right sensorimotor cortex and in the occipital cortex to estimate GABA concentrations (see methodology for the magnetic resonance spectroscopy acquisitions and analyses in [24]). Values reported in this manuscript are group means (± standard deviations).

### Genetic analysis

DNA was extracted from a peripheral blood sample using the Nucleospin® blood kit (Macherey Nagel, Hoerdt, France). Paired-end sequencing (2 × 75bp) was performed on a NextSeq500 (Illumina Inc., San Diego, CA, USA) after standard library preparation (SeqCap EZ, Roche, Pleasanton, CA, USA) targeting 268 genes previously implicated in neurodevelopmental disorders. Gene list is available in Supplementary material (Table S1). We used an in-house bioinformatics pipeline based on the GATK v3.5 best practice guidelines (https://software.broadinstitute.org/gatk/best-practices) and analyzed rare (minor allele frequency < 1%) non-synonymous, splice site and indel variants. We used ACMG/AMP guidelines for variant classification [25]. Sanger sequencing was used for confirmation and parental study.

## Results

### Behavioral and neuropsychological characterization

#### General information

The patient was a French 28-year-old man and the first-born child to unrelated Caucasian parents. He had two younger healthy sisters. There was no family history of developmental delay or ASD, even though his father was described to have poor social interactions and a very scheduled life. The patient presented with no particular diseases during childhood and early adulthood, and no history of seizures.

His clinical examination (at age 28), including a thorough neurological examination, was normal. He had no particular dysmorphic features. His hearing was considered normal according to the patient, but no specific hearing test was performed. The patient achieved higher education (bachelor level) and worked as a lab assistant.

Upon examination, several investigation tests were prescribed, including a plasmatic amino acid chromatography, a urine organic acid analysis, ammonemia, copper metabolism, creatine metabolism, a standard karyotype and an array CGH, which did not reveal any anomaly.

#### ADI-R

His ADI-R scores are presented in Table 1. These scores indicate how the patient performed in the areas of reciprocal social interactions, communication, restricted interests and repetitive behaviors, according to the parents of the patient.

**Table 1:** Characteristics of the ASD patient presenting with a *TBR1* pathogenic variant

		Patient		References	
ADOS	*Communication subscore*	4	↑	Threshold: 2	
	*Social interaction subscore*	8	↑	Threshold: 4	
ADI-R	*Social interactions*	18	↑	Threshold: 10	
	*Communication*	16	↑	Threshold: 8	
	*Restricted and repetitive behaviors*	4	↑	Threshold: 3	
Vineland-II	*Communication*	26	↓	48 (± 8) (< 1^st^ percentile)	
	*Daily life*	34	↓	70 (± 7) (2^nd^ percentile)	
	*Socialization*	31	↓	58 (± 8) (< 1^st^ percentile)	
WAIS-IV	*Verbal comprehension subscore*	122	↑	100 (93^rd^ percentile)	
	*Perceptual reasoning subscore*	88	↓	100 (21^st^ percentile)	
	*Working memory subscore*	83	↓	100 (13^th^ percentile)	
	*Processing speed subscore*	81	↓	100 (10^th^ percentile)	
Autism-spectrum quotient (AQ)				Controls	ASD
	*Total score*	23	↑	16.4 (± 6.3)	35.8 (± 6.5)
	*Social skills subscore*	2	↔	2.6 (± 2.3)	7.5 (± 1.9)
	*Attention switching subscore*	5	↔	3.9 (± 1.9)	8.0 (± 1.8)
	*Local details subscore*	7	↔	5.3 (± 2.3)	6.7 (± 2.3)
	*Communication subscore*	5	↑	2.4 (± 1.9)	7.2 (± 2.0)
	*Imagination subscore*	4	↑	2.3 (± 1.7)	6.4 (± 2.1)
Glasgow Sensory Questionnaire (GSQ)				Low AQ	High AQ
	*Total hypersensitivity score*	34	↑	21.9 (± 8.4)	46.9 (± 14.9)
	*Total hyposensitivity score*	24	↔	19.6 (± 8.0)	35.9 (± 12.4)

Reference scores from other studies: mean (±standard deviation). Contrary to the other assessments, the GSQ and AQ are self-reported questionnaires and the reference scores are taken from published studies (AQ: [20], GSQ: [23]) but are not normative data. In comparison with the mean of a control or low AQ group, ↑: patient score superior to the mean (+1 SD), ↔: patient score in the range (±1 SD), ↓ patient score inferior to the mean (-1 SD)

Concerns of the parents were raised when the patient was 1–2 years old, due to his refusal to feed. He started to walk at 11 months old and showed a normal motor development, but he did not use gestures to communicate. At 2 years old, he did not communicate feeling pain and never asked adults for help. He tended to stay isolated in a corner of the room. At 4–5 years old, he did not play games involving imagination or role-playing and did not initiate interactions with other children. He enjoyed learning new facts and developed a strong interest in mythology. He frequently showed hyper-reactivity to auditory stimulations. He could handle minor changes in his daily routine. He showed stereotypies when he was a child (e.g., swinging or bouncing), but does not show stereotypies anymore. He has never shown aggressiveness.

In terms of language development, he started to say a few words at 3 years old and presented with echolalia. From 40 months old, he started to develop speech after receiving regular speech therapy. At 4 years old, he could understand oral instructions to perform actions and understood more than 50 words. He made inversions between first and third persons. At 4–5 years old, he did not pay attention when someone was speaking to him. Learning to speak was long and difficult. As a teenager, he did not know how to use reciprocal talk and conversations were limited in terms of flexibility and topics. As an adult, he has a good understanding of simple language and does not show echolalia anymore. He is now able to make small talk and does not use stereotyped sentences. He is verbally fluent and can be easily understood by people, including those who do not know him. Yet, he has difficulties understanding figurative sentences or implicit meanings. He has never used motion and gestures to communicate (e.g., nodding). He has a monotone voice and only shows changes in prosody when he feels positive emotions. In terms of facial expressions, he mainly expresses happiness and pain.

#### Vineland-II

The Vineland-II evaluated the ability of the patient in the areas of communication, daily life and socialization. His scores are presented in Table 1. Overall, the patient showed a low adaptive ability (1^st^ percentile).

### Communication

In terms of receptive language, the patient shows good understanding and listening abilities. He can follow instructions but needs to write things down to avoid forgetting them. If the instructions are clear and if the action must be executed immediately, he does not need to use this strategy. In terms of expressive language, he has good language skills, but these are inferiors to what is expected for an adult. He can follow several conversations on different topics, but only if conversations do not last for too long (less than 10 min). He has difficulties explaining complex ideas. He can reach long-term objectives, given that he is provided with help to plan his project. In terms of written language, he has always had difficulties writing (e.g., he can write short sentences but no official mails). He has good reading skills.

### Daily life

He encounters a few difficulties in his daily life, but has lived by himself in his apartment and has managed his finances for several months. He now lives with his parents, but he is autonomous in managing his daily life. He needs assistance on special occasions (e.g., scheduling a medical appointment). He has several social skills for community life, but he can be limited by certain contexts (e.g., he gets anxious if he has to go to unfamiliar places).

### Socialization

He is relatively adapted in socialization, has friends and enjoys spending time with them. He took acting classes and performed in theatre plays. Yet, he has difficulties initiating or planning activities with his friends and is limited in communicating with them. He understands most emotions, but has difficulties understanding many signs of non-verbal communication (e.g., if someone yawns out of boredom). He has difficulties inferring that people do not know what he is thinking of and talking about the others’ interests. He acts in an appropriate and decent way and is usually very cautious. He manages to control his anger or pain.

### ADOS assessment

The patient scored above the threshold for ASD both at the communication and social interaction subscores of the ADOS [16] (Table 1). His language was assessed as being formal. His prosody was relatively monotonous and the sentence structures were somehow repetitive. The reciprocity of the conversation was not always well mastered. The patient did not use gestures while talking, and the patient was frequently avoiding eye contacts. His facial expressions remained neutral across the ADOS testing. Communicating about affects was difficult.

### WAIS assessment

The patient presented with good cognitive functions (no intellectual disability). He had heterogeneous scores both between and within domains (Table 1). The verbal comprehension subscore was in the superior average (93^rd^ percentile), while the perceptual reasoning, working memory and processing speed subscores were all in the low averages (10^th^ to 21^st^ percentiles). The scores of verbal comprehensions were good in the three main exercises: similitudes (14), vocabulary (13) and information (14). The optional exercise assessing social comprehension was the only score (8) below the average. The subscores of perceptual reasoning were heterogeneous: praxis functions were low, whereas analogic reasoning was good. The working memory assessment revealed weaknesses in short-term auditory verbal memory, whereas working memory was relatively efficient. The processing speed was in the low average, as it took time for the patient to elaborate a cognitive treatment in the visual and verbal modalities.

### Questionnaires assessing autistic traits and sensory sensitivity

The patient scored 23 (i.e., close to the range of the control group) at the AQ (Table 1), despite a higher number of autistic traits observed by clinicians than reflected by the self-assessed AQ.

The hypersensitivity score of the patient at the GSQ was higher than the average score of a low AQ group (Table 1). As compared to a low AQ group, the patient experienced more frequently hypersensitivity in the auditory, gustatory and proprioceptive modalities, and hyposensitivity in the visual and proprioceptive modalities. He reported less auditory and gustatory hyposensitivity than a low AQ group.

### Neuroimaging data

A careful visual inspection of the brain MRI scan did not reveal signs of abnormalities in brain morphology or in the demarcation between the grey and white matters (Supplementary material: Figure S1).

The patient’s GABA concentrations were 2.15 i.u. in the somatosensory cortex and 0.98 i.u. in the occipital lobe (Supplementary material: Figure S2). In comparison, GABA concentrations in these regions were, respectively, 2.38 (± 0.54) i.u. and 2.44 (± 0.59) i.u. in an ASD group (excluding the patient reported here) and 2.85 (± 0.86) i.u. and 2.22 (± 0.51) i.u. in a control group [24]. Using a one-sample *t* test, we found that the GABA measurements of this patient were significantly lower than those of the control (*t*(18) = 10.7, *p* < .0001) and ASD (*t*(16) = 9.9, *p* < .0001) groups in the occipital cortex, and lower than the mean of the control group in the somatosensory cortex (*t*(18) = 3.5, *p* <.01).

### Genetic results

Mean depth of sequencing was 247× and 97.2% of the target was covered at 30× minimum. Analysis revealed a heterozygous single base deletion within the *TBR1* gene (NM_006593.3:c.26del) leading to a frameshift and the creation of a premature stop codon: NM_006593.3:p.(Pro9Leufs*12). This variant is absent from the gnomAD resource aggregating data from large-scale sequencing projects [26]. Parental testing using Sanger sequencing showed that it occurred de novo. For all these reasons, this variant was classified as pathogenic.

## Discussion

This article describes the case study of an adult with ASD who has a de novo heterozygous single base deletion in *TBR1* leading to an early premature stop codon. This patient showed a classical phenotype of ASD without intellectual disability and with a slight language delay in early childhood. He had no structural brain abnormalities, but low concentrations in GABA.

### *TBR1* pathogenic variants in ASD

**Fig. 1:**
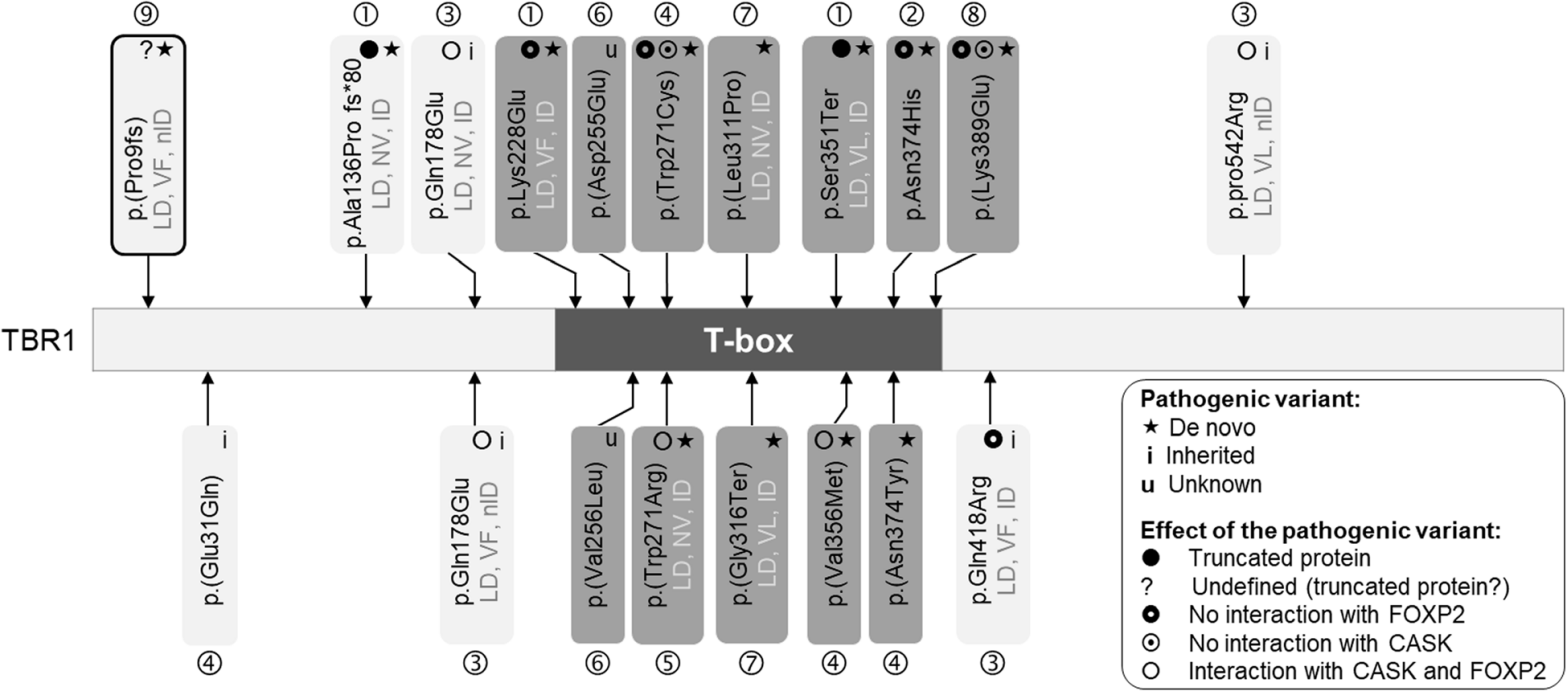
Pathogenic variants associated with ASD identified in the TBR1 protein. Predicted protein consequences of single-nucleotide variants found in *TBR1* in ASD, described on https://gene.sfari.org/database/human-gene/TBR1, in the following references [11, 27–34] and in the present study (circled in black). Pathogenic variants found in the T-box domain coding sequence are indicated in dark grey. LD: language delay, VF: verbally fluent, VL: verbally limited (few words or sentences), NV: non-verbal. The TBR1 protein consists of 682 amino acids. References: ① [28], ② [27], ③ [30], ④ [34], ⑤ [31], ⑥ [32], ⑦ [11], ⑧ [33], ⑨ Present study

Nineteen pathogenic variants have been described in *TBR1* in ASD patients (Fig. 1), including 16 missense variants and 3 truncating variants. In addition, heterozygous *TBR1* whole-gene deletions have also been reported [35]. Most of the studies investigating *TBR1* variations in ASD were performed in large cohorts and do not provide detailed phenotypes of the patients. In an attempt to fill this gap, a recent article detailed the clinical phenotype of a young girl with ASD who had a de novo pathogenic variant in *TBR1* [11]. The authors highlighted that among 10 ASD individuals with a *TBR1* pathogenic variant, 100% showed a language delay and 80% had intellectual disability [11]. Note that the *Tbr1*^+/−^ mouse models of ASD [36, 37] show impaired social interactions, cognitive flexibility, and associative memory, but the autistic symptoms can be decreased by D-cycloserine and clioquinol treatments [36, 38, 39].

Here, the pathogenic variant identified in *TBR1* possibly leads to the translation of a short truncated protein and/or to an unstable mRNA due to non-sense-mediated mRNA decay. Yet, very early premature termination codons, such as the one reported here, are sometimes associated with a reinitiation of translation and hence fail to trigger nonsense-mediated mRNA degradation [40, 41]. A marked reduction in mutant protein expression has been observed in these cases [40, 41]. Concerning *TBR1* c.26del, translation reinitiation could occur at p.Met12 as the corresponding ATG codon has a good Kozak consensus sequence and would then result in the translation of an N-terminally truncated TBR1 protein missing 11 amino-acids out of 682.

### *TBR1* and brain development

*TBR1* encodes T-box brain protein 1, a neuron-specific transcription factor of the T-box family that plays a major role in brain development [42–44]. *TBR1* ortholog in mice is expressed in the deep layers of the cerebral cortex, in the hippocampus, amygdala and olfactory bulb. It regulates neuronal differentiation and migration, and axonal guidance. TBR1 controls a transcriptional cascade that is thought to be relevant to the ASD pathogenesis [45, 46], as 24 target genes have been associated with ASD. TBR1 modulates key genes, such as *RELN* that is essential to neuronal migration and cortical layer organization [47]. Given the major role of the *TBR1* gene in brain development and the early frameshift pathogenic variant identified in the patient, we could expect a more severe phenotype. Indeed, a recent study showed that a de novo frameshift pathogenic variant in *TBR1* was associated with developmental encephalopathy [48], whereas here, the whole-brain MRI of the patient appeared to be typical. A reinitiation of translation could potentially account for the absence of visible brain abnormalities. Note that given the resolution of the MRI, we cannot draw conclusions about structural integrity on a smaller scale.

Importantly, *TBR1* is also involved in the regulation of early gene expression of GABAergic interneurons [49]. *TBR1* may also influence the relative number of glutamatergic and GABAergic neurons in the brain [42]. Indeed, *Tbr1* deficient mice show increased *Gad1* expression [45], a gene encoding the glutamate decarboxylase GAD67 that converts glutamate into GABA. Interestingly, GAD67 levels may be reduced in the cortex of individuals with ASD [50]. With an heterozygous *TBR1* frameshift pathogenic variant, the patient would therefore be expected to show an increase in GABA levels, whereas he showed decreased GABA levels in the occipital and somatosensory cortices. We can hypothesize that other causes such as immature GABA neurons could account for this decreased level [51]. Interestingly, artificially decreasing GABA action by using an antagonist of GABA_A_ receptors can induce *TBR1* expression in cultured neurons [42].

### *TBR1* and language development

TBR1 interacts with FOXP2, a transcription factor involved in language impairments [52]. Consequently, *TBR1* pathogenic variants altering or preventing the heterodimerization with FOXP2 should be associated with impaired language abilities. In ASD, most of the de novo pathogenic variants in *TBR1* are missense pathogenic variants in the T-box, which is the FOXP2 interacting sequence (Fig. 1). Such variants often lead to disrupted interactions with FOXP2. Two truncating pathogenic variants previously described in ASD [27–29] were located 5′ to the T-box coding domain in a non-verbal boy and in the T-box in a girl with limited language [30]. In contrast, in the present study, the patient was relatively fluent at 8 years of age and is now completely fluent as an adult. If the *TBR1* pathogenic variant described here indeed leads to translation reinitiation, it could explain why three de novo heterozygous pathogenic variants in *TBR1* were associated with very different outcomes in terms of language abilities (severe, mild or no language impairment).

### *TBR1* and intellectual disability

TBR1 binds to CASK, which is a membrane-associated guanylate kinase playing a key role in intellectual disability [30, 53]. Heterozygous loss-of-function mutations in the X-linked *CASK* gene have been associated with severe intellectual disability and with ASD [48, 54, 55]. The TBR1-CASK complex regulates the expression of genes involved in brain development [56] and of several candidate genes for ASD and intellectual disability. Truncated versions of TBR1 result in a loss of interaction between TBR1 and CASK, therefore affecting the expression of downstream target genes [30]. Pathogenic variants expected to lead to truncated TBR1 proteins were all associated with intellectual disability [11]. Surprisingly, here, the patient did not show intellectual disability, despite a frameshift pathogenic variant that should be associated with a truncated TBR1 protein. The alternative hypothesis of a translation reinitiation could be relevant as it may lead to a NH2-terminally truncated TBR1 protein able to interact with CASK. The TBR1-CASK complex levels may be decreased in this patient, which could explain why his WAIS scores for perceptual reasoning, working memory and processing speed were on average low. The only *TBR1* pathogenic variants described in individuals with ASD with normal intelligence were inherited missense pathogenic variants located outside the T-box and predicted to lead to normal interactions with CASK and FOXP2 [53].

### TBR1 and other symptoms of ASD

*TBR1* haploinsufficiency has been associated with impaired social interactions in mice models of ASD [37]. The patient described here also showed impairments in the social domain. One underlying mechanism could be that *TBR1* haploinsufficiency affects axonal projections of amygdalar neurons, leading to structural and functional abnormalities in the amygdala [37]. We can also note that three pathogenic variants of *TBR1* have been associated with a motor delay in ASD [11, 31], while the patient described in this study showed typical motor development.

### Limitations

Only very few people with ASD (less than 1%) present with a pathogenic variant in *TBR1* and the genetic architecture of ASD remains very complex. For instance, de novo pathogenic variants in ASD may not always account for the whole symptomatology, as the disease penetrance for identical twins of such mutations is inferior to 100% [57], but these variants could have additive effects. Importantly, the patient described in this case report may also carry other unidentified mutations that contributed to his phenotype. We refer to the literature about the *Tbr-* deficient mice models of ASD, but the comparison with animal models of ASD remains limited. Finally, we do not know whether the mutation described here leads to a very short TBR1 protein or to a translation reinitiation.

## Conclusions

The patient described here presents with a typical form of ASD with no comorbidities, despite the fact that he harbors a de novo pathogenic variant in *TBR1*. This variant introduces an early premature termination codon, which could either be associated with (1) no protein due to non-sense-mediated mRNA decay, (2) a short and C-terminally truncated protein unable to interact with proteins such as CASK or FOXP2, or (3) a N-terminally truncated protein due to a translation reinitiation that may lead to a (partially) functional protein of lower abundance than the wild-type protein. Further in vitro investigations of the effect of this pathogenic variant on translation reinitiation will be needed to shed light on this case study, but as this gene is expressed exclusively in the brain [30], a heterologous system will have to be used. In ASD, frameshift pathogenic *TBR1 *variants leading to premature stop codons occurring later in the coding sequence have been associated with intellectual disability and language delay. In contrast, the patient described here presented with normal intelligence, motor development and speech abilities. Other symptoms such as his language delay in early childhood or his social impairment could be caused by the *TBR1* pathogenic variant. A full phenotypic characterization of patients in whom ASD pathogenic variants have been identified is crucial in order to get more insight about the genetics of ASD and to better understand this disorder, which are prerequisites to providing accurate genetic counseling and adapted medical care to patients.

## Supplementary information

**Additional file 1: Table S1.** List of the 268 genes investigated. **Figure S1.** Whole-brain T1 anatomical scan of the patient. Frontal (left), sagittal (middle) and coronal (right) views of the anatomical scan of the patient described in the case report. **Figure S2.** GABA concentrations in occipital and somatosensory regions of interest. The patient reported in the case report is indicated by the dark red square. Data from ASD (orange) and neurotypical (NT – blue) groups (see [22]). iu: international unit. Somato: somatosensory

## Data Availability

All data relevant to the study are included in the article or uploaded as supplementary material. Any extra information can be asked to the authors.

## Abbreviations

AQ: Autism-spectrum quotient
ASD: Autism spectrum disorder
CASK: Calcium/calmodulin-dependent serine protein kinase
FOXP2: Forkhead box protein 2
GSQ: Glasgow Sensory Questionnaire
SNV: Single-nucleotide variation
TBR1: T-box brain 1
WAIS: Wechsler Adult Intelligence Scale
