# Supplementary material for: A de novo frameshift pathogenic variant in TBR1 identified in autism without intellectual disability
Source: Hum Genomics. 2020 Sep 18;14:32. doi: 10.1186/s40246-020-00281-5 (PMC7501624; doi:10.1186/s40246-020-00281-5)
Supplement: Supplementary file 1 — Additional file 1: Table S1. List of the 268 genes investigated. Figure S1. Whole-brain T1 anatomical scan of the patient. Frontal (left), sagittal (middle) and coronal (right) views of the anatomical scan of the patient described in the case report. Figure S2. GABA concentrations in occipital and somatosensory regions of interest. The patient reported in the case report is indicated by the dark red square. Data from ASD (orange) and neurotypical (NT – blue) groups (see [22]). iu: international unit. Somato: somatosensory [file 40246_2020_281_MOESM1_ESM.docx]

| ACSL4 | ASAH1 | CPS1 | GABRB3 | IKBKG | MMACHC | PDHA1 | SATB2 | SYNGAP1 |
| --- | --- | --- | --- | --- | --- | --- | --- | --- |
| ACTB | ASL | CREBBP | GALT | IL1RAPL1 | MTR | PDHX | SCN1A | SYP |
| ACTG1 | ASPM | CTNNB1 | GAMT | IQSEC2 | MUT | PEX12 | SCN2A | TBC1D24 |
| ACY1 | ASS1 | CTSF | GATAD2B | IVD | MYCN | PEX12 | SCN8A | TBL1XR1 |
| ADNP | ATP7A | CUL4B | GATM | KANSL1 | NAA10 | PEX6 | SETBP1 | TBR1 |
| ADSL | ATP7B | CUX2 | GBA | KCNJ10 | NAGLU | PEX7 | SETD5 | TBX1 |
| AFF2 | ATR | CYP27A1 | GCDH | KCNQ2 | NDST1 | PHF6 | SGSH | TCF4 |
| AGA | ATRX | CYP2U1 | GCH1 | KCNT1 | NDUFA1 | PHF8 | SHANK3 | TIMM8A |
| AHI1 | BCKDHA | DBT | GDI1 | KDM5C | NF1 | PHGDH | SKI | TPP1 |
| ALDH18A1 | BCKDHB | DCX | GK | KDM6A | NFIX | PIGV | SLC16A2 | TRAPPC9 |
| ALDH3A2 | BCKDK | DDHD2 | GLDC | KIAA2022 | NHS | PLP1 | SLC17A5 | TSC1 |
| ALDH5A1 | BCOR | DEAF1 | GPC3 | KIF1A | NIPBL | PMM2 | SLC25A13 | TSC2 |
| ALG1 | BRWD3 | DEPDC5 | CRIA3 | KMT2D | NLGN4X | PNKP | SLC25A15 | TSPAN7 |
| ALG12 | BTD | DHCR7 | CRIN1 | L1CAM | NPC1 | PORCN | SLC2A1 | TUBA1A |
| ALG13 | C12orf57 | DKC1 | CRIN2A | LAMC3 | NPC2 | PPP2R5D | SLC35A2 | TUBB2B |
| ALG3 | CASK | DLG3 | GRIN2B | LAMP2 | NRXN1 | PPT1 | SLC35C1 | TUSC3 |
| ALG6 | CBS | DPAGT1 | GUSB | MAN1B1 | NSD1 | PQBP1 | SLC6A1 | UBE2A |
| AMT | CDK5RAP2 | DPM1 | HCCS | MAN2B1 | NSDHL | PRODH | SLC6A8 | UBE3A |
| ANKRD11 | CDKL5 | DYNC1H1 | HCFC1 | MANBA | NSUN2 | PRPS1 | SLC9A6 | UBE3B |
| AP1S2 | CENPJ | DYRK1A | HDAC8 | MAOA | OCRL | PRRT2 | SMARCA2 | UPB1 |
| AP4B1 | CEP152 | EHMT1 | HEPACAM | MBD5 | OFD1 | PSAP | SMARCA4 | UPF3B |
| AP4E1 | CDH2 | EP300 | HEXA | MBTPS2 | OPHN1 | PTCHD1 | SMARCB1 | VLDLR |
| AP4M1 | CDH7 | FGD1 | HGSNAT | MCPH1 | OTC | PTEN | SMARCE1 | VPS13B |
| AP4S1 | CDH8 | FLNA | HPRT1 | MECP2 | PACS1 | PTPN11 | SMC1A | WDR45 |
| ARFGEF2 | CLCN2 | FMR1 | HRAS | MED12 | PAFAH1B1 | PTS | SMS | WDR62 |
| ARHGEF9 | CLN3 | FOXG1 | HSD17B10 | MED13L | PAH | QARS | SPTAN1 | WWOX |
| ARID1A | CLN5 | FOXP1 | IDS | MED17 | PAK3 | RAI1 | SRD5A3 | ZDHHC9 |
| ARID1B | CLN6 | FOXP2 | IDUA | MEF2C | PCCA | RBM10 | STIL | ZEB2 |
| ARSA | CLN8 | FTSJ1 | IGF1 | MFSD8 | PCCB | RELN | STXBP1 |  |
| ARX | CNTNAP2 | FUCA1 | IGF1R | MID1 | PCDH19 | RPS6KA3 | SYN1 |  |

**Supplementary Material**

**Table S1: List of the 268 genes investigated**


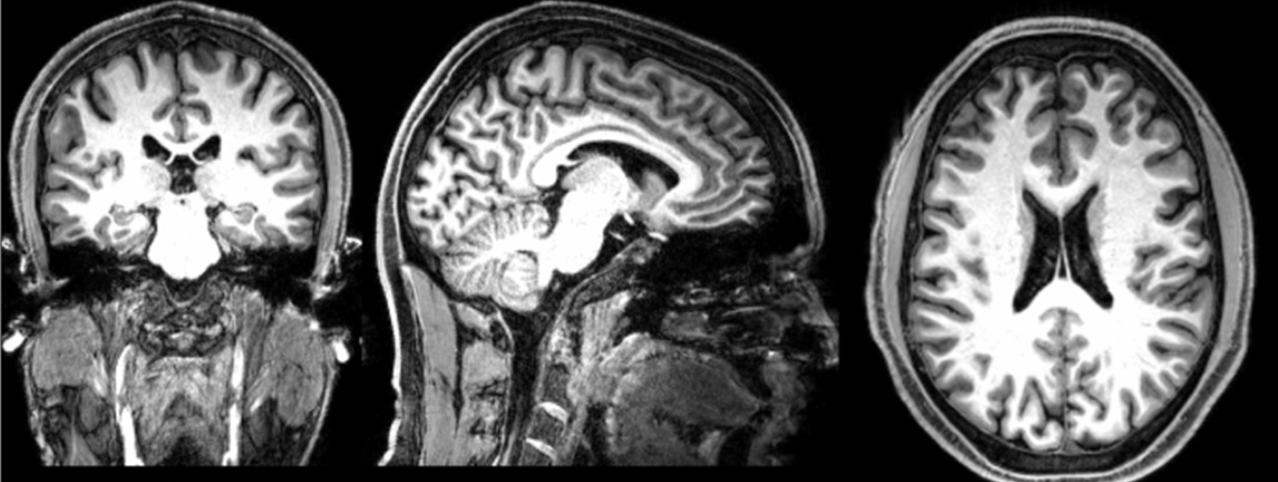


**Figure S1: Whole-brain T1 anatomical scan of the patient**

Frontal (left), sagittal (middle) and coronal (right) views of the anatomical scan of the patient described in the case report.

**
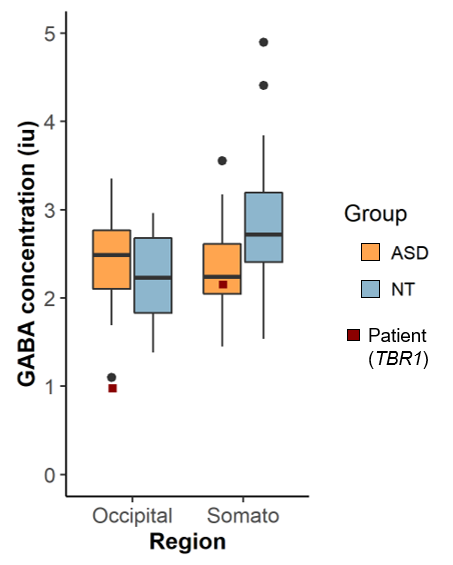
**

**Figure S2: GABA concentrations in occipital and somatosensory regions of interest**

The patient reported in the case report is indicated by the dark red square. Data from ASD (orange) and neurotypical (NT – blue) groups (see [24]). iu: international unit. Somato: somatosensory
